# Prevalence of gastrointestinal parasites in free-range yaks (*Bos grunniens*) in Gansu Province, Northwest China

**DOI:** 10.1186/s12917-019-2101-8

**Published:** 2019-11-15

**Authors:** Si-Yuan Qin, Ming-Yang Yin, Guang-Yao Song, Qi-Dong Tan, Jin-Lei Wang, Dong-Hui Zhou

**Affiliations:** 1 General Station for the Surveillance of Wildlife-borne Infectious Diseases, State Forestry and Grassland Administration, Shenyang, Liaoning Province 110034 People’s Republic of China; 2 State Key Laboratory of Veterinary Etiological Biology, Lanzhou Veterinary Research Institute, Chinese Academy of Agricultural Sciences, Lanzhou, Gansu Province People’s Republic of China; 3 Animal Quarantine Station of Beijing Entry-Exit Inspection and Quarantine Bureau, Beijing, 101312 People’s Republic of China; 4 Key Laboratory of Fujian-Taiwan Animal Pathogen Biology, College of Animal Sciences, Fujian Agriculture and Forestry University, Fuzhou, Fujian Province 350002 People’s Republic of China

**Keywords:** Prevalence, Gastrointestinal parasite, Yak, China

## Abstract

**Background:**

Little information about the prevalence of gastrointestinal parasites in yaks (*Bos grunniens*) in northwest China is available. Therefore, the objective of the study was to quantify faecal egg counts of gastrointestinal parasites (helminths and coccidia) in free-range yaks from Gannan Tibetan Autonomous Prefecture, Gansu Province, Northwest China.

**Results:**

Parasites were detected in 290 of 733 (39.56%) faecal samples. The results showed that Strongylidae, *Trichuris* spp*.* and *Eimeria* spp*.* were detected all year round, *Strongyloides papillosus* was detected in autumn and summer, and *Nematodirus* spp. was detected in both autumn and spring. In contrast, *Fasciola* spp. was only detected in spring. The prevalence rates of parasitic infections in different seasons were significantly different.

**Conclusions:**

To our knowledge, this is the first investigation of gastrointestinal parasites in yaks (*Bos grunniens*) in Gansu, China. The results demonstrated a high prevalence of gastrointestinal parasitic infections, specifically GN infections, in yaks in GTAP and these infections can cause economic losses to the local cattle industry.

## Background

In ruminants, gastrointestinal parasites, especially nematodes, can cause parasitic gastroenteritis (PG), which may lead to economic losses for livestock farmers [1]. The clinical symptoms of PG include weight loss, diarrhoea, anaemia and oedema. Livestock harbouring a diversity of parasite species will suffer severe symptoms [2]. The yak is a long-haired bovid found throughout the Himalayan region of southern Central Asia and the Tibetan Plateau and as far north as Mongolia and Russia. Most yaks are domesticated and are recognized as a major source of income for people in Gannan Tibetan Autonomous Prefecture (GTAP). The milk and meat of white yaks are popular delicacies for local Tibetan people and other residents in Gansu Province. However, it is very common that gastrointestinal parasitic infections cause a decline in milk production, weight loss and even death in yaks. Although some studies have reported the incidence of gastrointestinal helminthic infections in yaks in India [3–5] and in some provinces in China [6–10], there is little information about gastrointestinal parasitic infection in yaks in Gansu, China. To the best of our knowledge, this is the first study to estimate the prevalence of gastrointestinal parasites in yaks in the plateau areas of GTAP, Gansu, China.

## Results

The mean prevalence of gastrointestinal parasites in yaks is shown in Table 1. The Strongylidae and *Trichuris* spp*.* eggs were detected all year round. However, *Strongyloides papillosus* eggs were detected in only autumn and summer, *Nematodirus* spp. were detected in only autumn and spring, and *Fasciola* eggs were detected in only spring. The prevalence of *Eimeria* spp. in the different seasons ranged from 11.92 to 22.12%. A total of 39.56% (290) of the 733 faecal samples were positive for gastrointestinal parasite infection (Table 2). The prevalence of gastrointestinal parasites in yaks in the winter pastures (43.00%) was significantly higher than that in yaks in the summer pastures (35.11%) (*χ*^*2*^ = 4.69, *P* = 0.03) (Table 2). The prevalence of gastrointestinal parasites in yaks in the different seasons was 52.24% (autumn), 14.71% (winter), 58.93% (spring), and 8.61% (summer), and the prevalence rates were significantly different between the different seasons (*χ*^*2*^ = 134.197, *P* < 0.01).

**Table 1:** Prevalence of nematodes, trematode and protozoan in yaks in Gansu, China

Parasites	Autumn		Winter		Spring		Summer	
	No.positive/Prevalence (%)	95% CI^a^	No.positive/Prevalence (%)	95% CI	No.positive/Prevalence (%)	95% CI	No.positive/Prevalence (%)	95% CI
Nematodes								
Strongylidae	89.00/28.53	23.52–33.54	14.00/13.75	6.27–19.22	80.00/47.93	62.06–76.04	8.00/5.30	1.73–8.87
*Strongyloides papillosus*	19.00/6.09	3.44–8.70	ND^b^	–	ND	–	1.00/0.66	0.00–1.96
*Trichuris* spp*.*	5.00/1.60	0.21–3.00	1.00/0.98	0.00–2.89	20.00/11.91	7.01–16.80	3.00/1.99	0.00–4.21
*Nematodirus* spp*.*	69.00/22.12	17.51–26.72	ND	–	4.00/2.38	0.08–4.69	ND	–
Protozoan								
*Eimeria* spp*.*	69.00/22.12	17.51–26.72	15.00/14.71	7.83–21.58	23.00/13.69	8.49–18.89	18.00/11.92	6.75–17.09
Trematode								
*Fasciola* spp.	ND	–	ND	–	11/6.55	2.81–10.29	ND	–

^a^ Confidence intervals

^b^ Not detected

**Table 2:** Prevalence of gastrointestinal parasites infection in different pastures

Pastures	No. tested	No. positive	Prevalence (%)	95% CI^a^
Winter pastures^b^	414	178	43.00	38.23–47.76
Summer pastures^c^	319	112	35.11	29.87–40.35
Total	733	290	39.56	36.02–43.10

^a^ Confidence intervals

^b^ Autumn and winter

^c^ Spring and summer

*χ*^*2*^ = 4.69, *P* = 0.03

The mean EPG of Strongylidae and *Nematodirus* spp. and the mean OPG of *Eimeria* spp*.* in the different seasons are shown in Table 3. Interestingly, there were no Strongylidae and *Nematodirus* spp*.* collected in winter and summer. The mean EPG of Strongylidae in autumn and spring were 27.0 and 70.7, respectively. Furthermore, the mean EPG of *Nematodirus* spp. was lower in autumn (47.2) than in spring (50.0), and the mean OPG of *Eimeria* spp*.* in winter (26.7) and summer (66.7) were higher than those in autumn (15.9) and spring (13.0).

**Table 3:** Means of epg of gastrointestinal parasites in different seasons

Parasites	Autumn			Winter			Spring			Summer		
	Mean^a^	SE^b^	Min.–Max.^c^	Mean	SE	Min.–Max.	Mean	SE	Min.–Max.	Mean	SE	Min.–Max.
Strongylidae	31.4	±8.3	0.0–400.0	ND^d^	–	–	97.6	±16.6	0.0–700.0	25.0	±25.0	0.0–200.0
*Nematodirus* spp*.*	47.2	±10.5	0.0–400.0	ND	–	–	50.0	±28.9	0.0–100.0	ND	–	–
*Eimeria* spp*.*	15.9	±7.6	0.0–400.0	26.7	±15.3	0.0–200.0	13.0	±7.2	0.0–100.0	44.4	±27.1	0.0–400.0

^a^ Mean epg

^b^ Standard error

^c^ *Min*. minimum ranges, *Max*. maximum ranges

^d^ Not detected

## Discussion

The total prevalence of gastrointestinal parasite infection in yaks was 39.56%, which was lower than that in India (91.1%) [5] but higher than the multiple parasitic infection rate (7.47%) in yaks in North Sikkim, India [4]. The difference in the parasite prevalence may result from differences in immune capacities, feeding conditions, ecological conditions, temperature conditions, sampling seasons and animal welfare conditions.

The prevalence rates of gastrointestinal parasite infections in autumn, winter, spring and summer were 52.24, 14.71, 58.92 and 8.61%, respectively. The results of the chi-square test showed that the differences in the prevalence of gastrointestinal helminthic infections in the different seasons were significant (*P* < 0.05). Relevant research indicated that the prevalence of parasitic infection in spring was the highest, which was in accordance with the results of this study [3]. The reason for the different prevalence rates in the different seasons is as follows. The inhibitory larvae begin to grow when the weather becomes warm in early spring. After several days of growth, there is a peak period of ovulation. In summer, the number of eggs decrease because the ovulation of adult helminths is reduced. However, there are large numbers of L3 larvae in the grassland, and they may infect the yaks. For example, *Ostertagia ostertagi* can develop into L3 larvae in approximately 5–6 days at 25 °C [11]. The larvae can exit the faeces and protect themselves. The surviving larvae contaminate the grass and soil [12]. Then, there is a new peak period of ovulation in autumn as the L3 larvae become adults. The worm die of old age during the winter and the egg output declines accordingly [13]. In addition, the winter temperature in GTAP decreases significantly, which has a negative effect on the growth of gastrointestinal parasites.

The yaks are grazed on different pastures in winter and summer in GTAP. The statistical analysis indicated that the difference in the gastrointestinal helminthic infection rates between animals in the winter pastures and animals in the summer pastures was significant (*P* < 0.05), suggesting that the risk for gastrointestinal helminthic infection was higher in winter pastures than in summer pastures.

The EPG of gastrointestinal nematodes ranged from 0 to 700. The EPG of both Strongylidae and *Nematodirus* spp*.* in winter were 0, and the EPG of *Nematodirus* spp*.* in summer was 0, suggesting that the intensity of infection was not relatively high. *Eimeria* spp*.* was detected in each season, and the total prevalence was 17.05% (not shown), which was lower than that in Qinghai-Tibet and in Kenya [14, 15]. This is the first report on the dynamics of *Eimeria* spp*.* prevalence in yaks in China; however, further studies are needed to explain the difference above.

## Conclusions

This study investigated gastrointestinal parasitic infections in free-range yaks in Gansu, China, for the first time. The results demonstrated a high prevalence of gastrointestinal parasitic infections, specifically GN infections, in yaks in GTAP and these infections can cause economic losses for the local cattle industry in this area. The results of this study can help yak herders and veterinary professionals take action to prevent and control parasitic infections based on the prevalence of parasitic infections in different seasons and areas and the species of parasites to reduce parasitic infection burdens and increase productivity in the yaks.

## Methods

### Study area

This study was carried out from October 2013 to July 2014 in GTAP, which is located in the south of Gansu Province, China. The latitude and longitude of the high plateau area are 33°06′-34°33′ N and 100°46′-102°29′ E, respectively, while the altitude is between 3300 and 4806 m. The average annual temperature is 1.1 °C.

### Faecal samples

A total of 733 samples were collected during the four seasons of the year, and the number of samples collected in each season was as follows: autumn (312), winter (102), spring (168), and summer (151). Faecal samples were collected randomly from winter pastures (from October to March) and summer pastures (from April to September), including 8 farms according to the protocol of a previous study with some modifications [16]. Briefly, approximately 50 g fresh samples were collected from the floor immediately after animal defecation; the samples were put into disposable plastic gloves marked with locations, serial numbers and collection dates. Samples were placed in ice boxes and transported to the laboratory, then stored in refrigerators at 4 °C and processed as soon as possible.

### Coprological examination

#### Centrifugation

Two grams of a fresh faecal sample was added to water 5 times its volume, and the faecal and water were mixed equally. Each dilution was poured through a wire mesh screen with an aperture of 350 μm, and the liquid was collected. The liquid was centrifuged for 2 min at 3000 rpm, and the supernatant was poured off and discarded. Precipitates were used for smearing to detect trematode and cestode eggs. Based on our previous experiments, the result was not good using raw fecal samples by sugar flotation. So we used precipitated fecal samples by taking the extra step of the water wash in the sugar floatation method.

#### Sugar floatation method

The precipitates above were resuspended with 30 mL of Sheather’s sucrose solution and centrifuged for 5 min at 3000 rpm according to a previous report [17]. Iron loops were used to create a smear to detect nematode eggs and coccidial oocysts.

#### Faecal egg counts

The modified McMaster technique was used to count nematode eggs and coccidial oocysts in fresh faecal samples. An animal was recognized as severely infectied when faecal egg counts exceeded 500 eggs per gram of faeces (EPG) [18].

### Statistical analysis

The mean prevalence and confidence intervals were determined for gastrointestinal parasites, and the mean EPG and oocyst per gram of faeces (OPG), minimum and maximum ranges and standard errors were determined for gastrointestinal nematodes (GNs) and *Eimeria* spp*.* using the analytic software (PASW®) Statistics 18. The chi-square test was performed for GN collected in different seasons and from different pastures using the general linear model procedure with the statistical software.

## Data Availability

The datasets used and analyzed during the study are available from the corresponding author on reasonable request.

## Abbreviations

EPG: Eggs per gram
GN: Gastrointestinal nematode
GTAP: Gannan Tibetan Autonomous Prefecture
OPG: Oocyst per gram
PG: Parasitic gastroenteritis
